# Evidence for Human Norovirus Infection of Dogs in the United Kingdom

**DOI:** 10.1128/JCM.02778-14

**Published:** 2015-05-14

**Authors:** Sarah L. Caddy, Alexis de Rougemont, Edward Emmott, Laila El-Attar, Judy A. Mitchell, Michael Hollinshead, Gael Belliot, Joe Brownlie, Jacques Le Pendu, Ian Goodfellow

**Affiliations:** a Division of Virology, Department of Pathology, University of Cambridge, Addenbrookes Hospital, Cambridge, United Kingdom; b Section of Virology, Faculty of Medicine, Imperial College London, London, United Kingdom; c National Reference Centre for Enteric Viruses, Laboratory of Virology, University Hospital of Dijon, University of Bourgogne, Dijon, France; d Department of Pathology and Pathogen Biology, The Royal Veterinary College, Hatfield, Hertfordshire, United Kingdom; e INSERM, U892, CNRS UMR 6299, Université de Nantes, Nantes, France

## Abstract

Human noroviruses (HuNoVs) are a major cause of viral gastroenteritis, with an estimated 3 million cases per year in the United Kingdom. HuNoVs have recently been isolated from pet dogs in Europe (M. Summa, C.-H. von Bonsdorff, and L. Maunula, J Clin Virol 53:244–247, 2012, http://dx.doi.org/10.1016/j.jcv.2011.12.014), raising concerns about potential zoonotic infections. With 31% of United Kingdom households owning a dog, this could prove to be an important transmission route. To examine this risk, canine tissues were studied for their ability to bind to HuNoV *in vitro*. In addition, canine stool samples were analyzed for the presence of viral nucleic acid, and canine serum samples were tested for the presence of anti-HuNoV antibodies. The results showed that seven different genotypes of HuNoV virus-like particles (VLPs) can bind to canine gastrointestinal tissue, suggesting that infection is at least theoretically possible. Although HuNoV RNA was not identified in stool samples from 248 dogs, serological evidence of previous exposure to HuNoV was obtained in 43/325 canine serum samples. Remarkably, canine seroprevalence for different HuNoV genotypes mirrored the seroprevalence in the human population. Though entry and replication within cells have not been demonstrated, the canine serological data indicate that dogs produce an immune response to HuNoV, implying productive infection. In conclusion, this study reveals zoonotic implications for HuNoV, and to elucidate the significance of this finding, further epidemiological and molecular investigations will be essential.

## INTRODUCTION

Human noroviruses (HuNoV) are a major cause of viral gastroenteritis worldwide, with an estimated 3 million cases each year in the United Kingdom alone (1). HuNoV are members of the Caliciviridae family, which have a single-stranded positive-sense RNA genome and can cause a variety of disease manifestations in a wide range of species. The Norovirus genus itself is divided into at least six different genogroups based on capsid sequences (2, 3). HuNoV strains fall into genogroups I, II, and IV (GI, GII, and GIV). GII strains are responsible for 96% of HuNoV cases worldwide, with GII.4 genotypes being the most prevalent overall (4). In humans, HuNoV typically causes acute diarrhea, vomiting, and abdominal cramps, with the illness lasting on average 28 to 60 h (5). Infection is most common in health care institutions such as hospitals and long-term-care facilities (6), but outbreaks are often reported in association with schools, restaurants, cruise ships, and other settings such as military bases (7).

Transmission of HuNoV is via contact with feces or vomit, which occurs predominantly through direct person-to-person contact or contaminated food and water (8). Zoonotic transmission of HuNoV has also been proposed as a hypothetical route of infection (9). Both cattle and pigs have come under scrutiny for their potential role in transmitting HuNoV over the past decade. This has been precipitated by the identification of GII.4 HuNoV RNA in the stools of farmed pigs and cattle (10, 11). Furthermore, over half of the pigs in a U.S. report were seropositive to both GI and GII human noroviruses (12). This finding was supported by a study that demonstrated that human strains can replicate and induce an immune response in gnotobiotic pigs (13).

Dogs were first suggested to be potential zoonotic vectors of HuNoV in 1983, following an outbreak of norovirus gastroenteritis in an elderly-care home (14). Just prior to development of clinical symptoms in humans, the owner's dog was sick on multiple occasions around the home. Serological testing of the dog later revealed a moderate titer to HuNoV antigen by electron microscopy, whereas control dogs were all seronegative. Later evidence linking dogs with HuNoV infections in humans followed in an epidemiological study that showed that seropositivity to HuNoV in humans was higher if there was a dog in the household (15), and anti-HuNoV antibodies have recently been identified in dogs across Europe (16).

In 2012 it was reported that HuNoV could be detected in stool samples from pet dogs (17). Samples were collected from 92 dogs if the dog or owner had recently suffered from diarrhea or vomiting. Canine stool samples were tested for the presence of GI, GII, and GIV HuNoV, and 4 dogs were found to be positive for GII HuNoV. In one case, the HuNoV strain identified was identical to that isolated from stools of the owner. While the presence of identical sequences does not formally confirm active replication in dogs, the levels of viral RNA observed would suggest that at least limited replication had occurred.

The primary step for HuNoV infection of cells requires HuNoV binding to complex carbohydrates known as histo-blood group antigens (HBGAs) (18). As well as being expressed on erythrocytes, HBGAs are expressed on the surface of epithelial cells of the gastrointestinal, genitourinary, and respiratory tracts and can be secreted by these cells into bodily fluids, including saliva (19). Internalization of viral particles into cells occurs following HuNoV attachment to HBGAs *in vitro*, and therefore it has been proposed that the primary step for HuNoV uptake into cells is HuNoV binding to the HBGAs. The importance of HBGA binding in human infections has been demonstrated by experimental challenge studies. These have shown that susceptibility to HuNoV infection is related to expression of HBGAs in the gastrointestinal tract (20, 21). Approximately 20% of Caucasians do not express gastrointestinal HBGAs due to the lack of a functional fucosyltransferase 2 (*FUT2*) gene (“nonsecretors”), and consequently these individuals have a significantly reduced susceptibility to infection with noroviruses.

For dogs to be susceptible to human norovirus, it is reasoned that dogs must express HBGAs in their gastrointestinal tracts. Although canine blood types bear no resemblance to the human system, and indeed canine erythrocytes cannot be agglutinated by HuNoV (22), we have recently demonstrated that dogs do express HBGAs in their saliva and on the surface of intestinal epithelial cells (23). This indicates that dogs express the relevant attachment factors for the primary step in HuNoV infection, which is indicative of a theoretical susceptibility to HuNoV.

With approximately 10 million dogs in the United Kingdom, divided among 31% of the households (24), the suggestion that HuNoV may be transmissible between these species is of considerable public health concern. This study aimed to investigate the ability of HuNoV to infect dogs and the frequency with which this might be occurring in the United Kingdom. This has been achieved by first exploring the relationship between canine HBGA expression and HuNoV binding to canine tissues, and second by determining the occurrence of current and past HuNoV infections in dogs using an HuNoV RNA survey of canine stool samples and a serological survey of canine serum.

## MATERIALS AND METHODS

### Ethics statement.

Collection of canine saliva samples was a nonregulated procedure, and hence ethical approval was not required. Similarly, no ethical approval was required for collection of canine stool and serum samples, as these were either animal waste products, surplus to diagnostic requirements, or derived from a previously published and ethically approved study (25). Canine gastrointestinal tissue samples were donated by a large pharmaceutical company; the six dogs had been bred for scientific research but were deemed unsuitable for this purpose and were humanely euthanized. Human saliva samples were collected as part of a previous study (26), approved by the Nantes University Hospital Review Board (study no. BRD02/2-P), with informed written consent obtained from all saliva donors.

### Samples.

Stool samples were collected from dogs admitted to six participating United Kingdom veterinary clinics in Suffolk, Kent, Lincolnshire, Middlesex, and Cambridgeshire. Dogs were recruited to the study if they passed stools while hospitalized, and with owner consent, stools were collected by veterinary personnel. An additional 10 samples were collected from Wood Green Animal Shelter, Cambridgeshire, United Kingdom, from dogs suspected to have infectious gastroenteritis. All stool samples were stored at −20°C until and during transportation to the laboratory, whereafter they were stored at −80°C prior to extraction of RNA. Control stool samples from non-veterinary patients were collected from healthy dogs owned by veterinary staff, as well as from dogs at participating boarding kennels. Basic case data were recorded for each dog from which a stool sample was collected, including age, breed, sex, reason for admission, and any recent history of enteric disease.

Canine serum samples were obtained from two separate dog populations. Samples from 1999 to 2001 were collected from a rehoming kennel as part of an existing study (25). Serum samples from 2012 to 2013 were surplus to diagnostic requirements, obtained from veterinary patients at the Royal Veterinary College, London, from which blood was collected for biochemical analysis for diagnostic purposes.

Canine saliva samples were collected from 23 dogs at Wood Green Animal Shelter, Huntingdon, United Kingdom (numbered 1 to 23), and a further 3 samples were collected from three of the dogs at a pharmacological research company in the United Kingdom (labeled D, E, and F). The dogs at the animal shelter were mixed breeds, whereas the research dogs were beagles. Sample collection was achieved using a children's swab (Salimetrics, Newmarket, United Kingdom), from which saliva was extracted. Human saliva samples were collected as part of a previous study (26).

Canine tissue samples were donated from six healthy 18-month-old female dogs (labeled A to F) that had been humanely euthanized as surplus to industry research requirements. Sections of the gastrointestinal tract (1 cm^2^) were dissected as previously described (23). Briefly, either samples from the duodenum, jejunum, ileum, cecum, and colon were fixed and then embedded in paraffin and sectioned, or sections were lysed and homogenized to generate scraping samples.

### RNA extraction and reverse transcription-quantitative PCR (qRT-PCR).

Stools were diluted 10% (wt/vol) in phosphate-buffered saline (PBS) (pH 7.2), and solids were removed by centrifugation at 8,000 × *g* for 5 min. Viral nucleic acid was extracted from 140μl of each clarified stool suspension with the GenElute mammalian total RNA miniprep kit (Sigma-Aldrich) according to the manufacturer's instructions.

An internal extraction control was added to each sample during nucleic acid extraction to verify removal of PCR inhibitors and enable precise quantification of viral nucleic acid. A fixed amount of equine arteritis virus (EAV) RNA was added with the lysis buffer to each sample to obtain an EAV concentration of approximately 1 × 10^8^ copies per ml of fecal suspension. qRT-PCR was used to screen for genogroup I (GI) and genogroup (GII) HuNoV using previously published primer-probe sets (27). Samples were also screened for canine-specific noroviruses using a primer-probe set designed to identify six different strains of canine norovirus (CNV) (Table 1) as well as canine parvovirus (CPV) and canine enteric coronavirus (CECoV) in a duplex assay as previously reported (28).

**TABLE 1:** Primers and probe sequences used in qPCR screen of canine stool samples for noroviruses

Virus	Primer and probe sequences^*a*^	Reference
Canine norovirus	Forward, GCTGGATGCGGTTCTCTGAC; reverse, TCATTAGACGCCATCTTCATTCAC; probe, FAM-AGCGAGATTGCGATCTCCCTCCCACAT-BHQ	28
Human GI norovirus	Forward, CGYTGGATGCGNTTYCATGA; reverse, CTTAGACGCCATCATCATTYAC; probe, FAM-AGATYGCGATCYCCTGTCCA-TAMRA	27
Human GII norovirus	Forward, CARGARBCNATGTTYAGRTGGATGAG; reverse, TCGACGCCATCTTCATTCACA; probe, FAM-TGGGAGGGCGATCGCAATCT-TAMRA	27
Equine arteritis virus (internal control)	Forward, CATCTCTTGCTTTGCTCCTTAG; reverse, AGCCGCACCTTCACATTG; probe, Cy5.5-CGCTGTCAGAACAACATTATTGCCCAC-BHQ2	51

a FAM, 6-carboxyfluorescein; TAMRA, 6-carboxytetramethylrhodamine.

Using a 1-step qRT-PCR protocol, 2μl of extracted RNA was added to 2× Precision OneStep qRT-PCR MasterMix (PrimerDesign Ltd.), 6 pmol/μl primers, and 3 pmol/μl probe. The thermal cycle protocol, used with a ViiA7 qPCR machine (AB Applied Biosystems), was as follows: 55°C for 30 min, inactivation of reverse transcriptase at 95°C for 5 min, and then 40 cycles consisting of denaturation at 95°C for 15 s and then annealing and elongation at 60°C for 1 min.

### VLP production.

**FIG 1:**
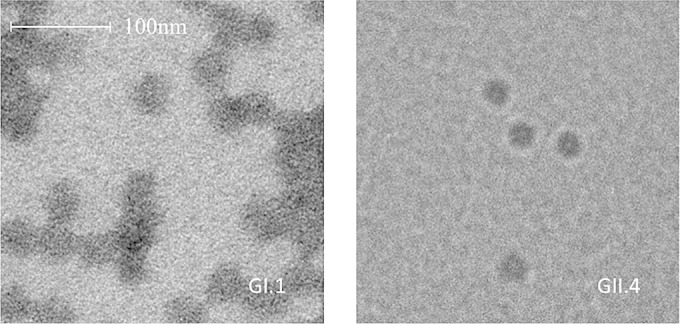
Characterization of HuNoV VLPs. Electron micrographs of representative GI and GII HuNoV VLPs (GI.1 and GII.4) with negative staining are shown.

Virus-like particles (VLPs) of seven different HuNoV genotypes (GI.1, GI.2, GI.3, GII.3, GII.4, GII.6, and GII.12) and VLPs of three strains of CNV were produced using a previously described method (28–30). Accession numbers for the HuNoV strains used to generate the VLPs for this study are listed in Table 2. Recombinant baculoviruses containing human or canine norovirus VP1 protein were generated, and then VLPs were produced by infection of Hi5 insect cells. VLPs were released from the infected Hi5 cells by freeze-thawing and then clarified by removing cellular debris (6,000 × *g*, 30 min) and baculovirus (14,000 × *g*, 30 min). VLPs were partially purified through a 30% (wt/vol) sucrose cushion in TNC buffer (50 mM Tris HCl [pH 7.4], 150 mM NaCl, 10 mM CaCl_2_) containing the protease inhibitor leupeptin at 150,000 × *g* for 2 h. The pelleted VLPs were resuspended in TNC and further purified by isopycnic centrifugation in cesium chloride (150,000 × *g*, 18 h). The resultant VLP bands were collected by puncture, and the solution containing VLPs was dialyzed against PBS prior to quantification by the bicinchoninic acid (BCA) protein assay (Thermo Scientific) and storage at −80°C. GI.1 and GII.4 VLPs were visualized by electron microscopy to confirm correct particle assembly (Fig. 1), and as all VLPs in this study were made using an identical protocol and formed a defined band on a cesium chloride gradient, this was deemed sufficient to confirm VLP formation for each genotype.

**TABLE 2:** GenBank Accession numbers of HuNoV strains used to generate VLPs

HuNoV genotype	GenBank Accession no.
GI.1	NC_001959.2
GI.2	KP064095
GI.3	KP064096
GII.3	KP064097
GII.4	AF472623
GII.6	KP064098
GII.12	KP064099

### Enzyme-linked immunosorbent assay (ELISA) procedure.

Ninety-six-well polystyrene microtiter plates (Nunc Maxisorb; Fisher Scientific) were coated overnight at 4°C with 25 ng of each GI strain (3 strains, total of 75 ng/well) or each GII strain (4 strains, total of 100 ng/well) in 0.05 M carbonate-bicarbonate buffer (pH 9.6). Plates were washed three times with 0.05% Tween 20 in phosphate-buffered saline (PBS-T) before blocking in 5% skim milk–PBS-T for 1 h at 37°C and then three PBS-T washes. Plates were then incubated for 3 h at 37°C with a 1:50 dilution of each serum sample in duplicate in 5% skim milk–PBS-T. Pooled human sera (Sigma-Aldrich), diluted 1:400, and 100 ng pooled GII human norovirus VLPs were used as a positive control. After three washes with PBS-T, 50 μl of horseradish peroxidase (HRP)-conjugated anti-canine or anti-human IgG antibody (both from Sigma-Aldrich) diluted 1:5,000 or 10,000, respectively, in 5% milk–PBS-T was added to each well and incubated at 37°C for 1 h. The plates were washed three times with PBS-T and bound antibody detected with 50 μl tetramethylbenzidine (TMB) (Sigma-Aldrich), followed by incubation at room temperature for 10 min. The reaction was stopped with 1 N H_2_SO_4_, and the optical density at 450 nm (OD_450_) was read (Spectromax M2 plate reader; Molecular Devices).

To eliminate the possibility that nonspecific components of the VLP preparation were identified by the canine sera, an antigenically distinct vesivirus 2117 VLP was included in the assay. The OD_450_s of serum samples incubated on either carbonate-bicarbonate buffer-coated wells or vesivirus 2117-coated wells were highly comparable, with the exception of 8% of dogs which displayed reactivity to vesivirus 2117, which was a limitation of this methodology (data not shown). It was suspected that reactivity to vesivirus 2117 could be due to cross-reactivity with the related canine calicivirus, but no correlation between seropositivity to HuNoV or seropositivity to vesivirus 2117 was shown (see Fig. S1 in the supplemental material). Subsequently, the background signal for each sample was determined by measuring the OD_450_ of serum samples incubated with carbonate-bicarbonate buffer alone. The background signal was then subtracted from the OD_450_ of VLP-coated wells to generate the corrected OD_450_ value. A threshold value was established as the mean of the OD_450_s of all buffer-coated cells plus 3 standard deviations. A serum sample was considered positive when the corrected OD_450_ was higher than the threshold. Any serum samples showing a positive response to pooled HuNoV VLPs were subjected to further testing with individual HuNoV VLPs. Plates were coated with 25 ng of individual VLPs in carbonate-bicarbonate buffer and the protocol then repeated as described above.

Evaluation of serological cross-reactivity between different norovirus strains was achieved using VLP competition assays and antibody competition assays. For the VLP competition assays, plates were first coated with 25 ng/well of VLP overnight at 4°C. Canine serum was incubated with a range of concentrations of either pooled GI or GII HuNoV VLPs or pooled CNV VLPs (0.5, 1, 2, and 4 μg/ml) for 1 h at 37°C. Vesivirus 2117 VLP was incubated with the canine sera as a negative control. After the incubation period, 50 μl of each serum-VLP combination was added to the previously VLP-coated plates. The remainder of the ELISA protocol was followed as detailed above. The concentration of VLP required to block 50% binding (50% effective concentration [EC_50_]) was calculated by fitting sigmoidal curves to the serial dilution data. Samples unable to block 50% of binding at the highest dilution tested were assigned an EC_50_ of 2.5× the assay upper limit of detection.

For the antibody competition assays, polyclonal anti-norovirus VLP antibodies were generated by immunization of a rabbit (GII.4 HuNoV) or rat (CNV) as previously described (31). Plates were coated with 25 ng/well of GII.4 VLP overnight at 4°C, and then after blocking for 1 h in 5% skim milk–PBS-T, rabbit anti-GII.4 or rat anti-CNV antibody was added to the wells serially diluted in 5% skim milk–PBS-T for 1 h. Following three washes in PBS-T, GII.4-positive canine serum was added and the remainder of the ELISA protocol followed as described above.

Assays to assess VLP binding to saliva and gastrointestinal scrapings used the ELISA protocol as described above, with the addition of 100 ng HuNoV VLPs per well in 5% skim milk–PBS-T after the 1-h blocking step with 5% skim milk–PBS-T. VLPs were incubated at 37°C for 1 h with the saliva or gastrointestinal scraping samples and then detected using polyclonal anti-GI.1 (rabbit 130) or anti-GII.4 (rabbit 132) primary antibodies. Goat HRP-conjugated anti-rabbit antibody (Interchim, France) was used as the secondary antibody as previously described. The saliva phenotyping assay used the ELISA protocol as detailed above, with variations as described in a previous study (23).

### SDS-PAGE and Western blot analysis.

VLPs were heated to 95°C for 5 min in the presence of SDS loading buffer and electrophoresed on 12.5% SDS-polyacrylamide gels. For Coomassie blue staining, the gels were incubated with Coomassie blue for 1 h at room temperature prior to destaining. Proteins were transferred from SDS-polyacrylamide gels to polyvinylidene difluoride membranes for Western blotting. The membranes were blocked for 1 h at room temperature with 5% milk in PBS-T and then incubated overnight at 4°C with canine serum samples diluted 1:1,000. The excess antibody was washed three times in PBS-T and incubated for 1 h with anti-canine IgG secondary antibody conjugated to horseradish peroxidase (Sigma-Aldrich) diluted 1:10,000 in 5% milk–PBS-T. After washing away excess secondary antibody, the bands were detected using enhanced chemiluminescence reagent (GE Healthcare).

### Tissue samples and immunohistochemical analysis.

Tissue sections from the gastrointestinal tracts of six dogs were deparaffinized through baths of LMR-SOL (1-bromopropane, 2-methylpropane-2-ol, and acetonitrile), followed by rehydration with successive baths of 100, 90, 70, and 50% ethanol. Endogenous peroxidase activity was blocked with 0.3% hydrogen peroxide in PBS. Nonspecific binding was blocked with 3% bovine serum albumin (BSA) in PBS. H and A antigen detection was then performed as previously reported (23). To assess the ability of VLPs to bind to tissue sections, after blocking, 1 μg/ml VLPs was incubated with the sections overnight at room temperature. Anti-HuNoV primary antibody was then incubated with the tissue sections for 1 h at 37°C. After three washes in PBS, sections were incubated with secondary anti-rabbit biotinylated antibody (Vector Laboratories, Burlingame, CA) diluted in 1% BSA in PBS for 1 h. Sections were washed three times in PBS prior to addition of HRP-conjugated avidin D (Vector Laboratories, Burlingame, CA) also diluted in 1% BSA in PBS. Substrate was added to the slides (AEC kit; Vector Laboratories, Burlingame, CA), followed by Mayer's hematoxylin solution (Merck, Whitehouse Station, NJ) for contrast staining.

## RESULTS

### HuNoV VLPs bind to canine gastrointestinal samples in ELISA-based assays.

**FIG 2:**
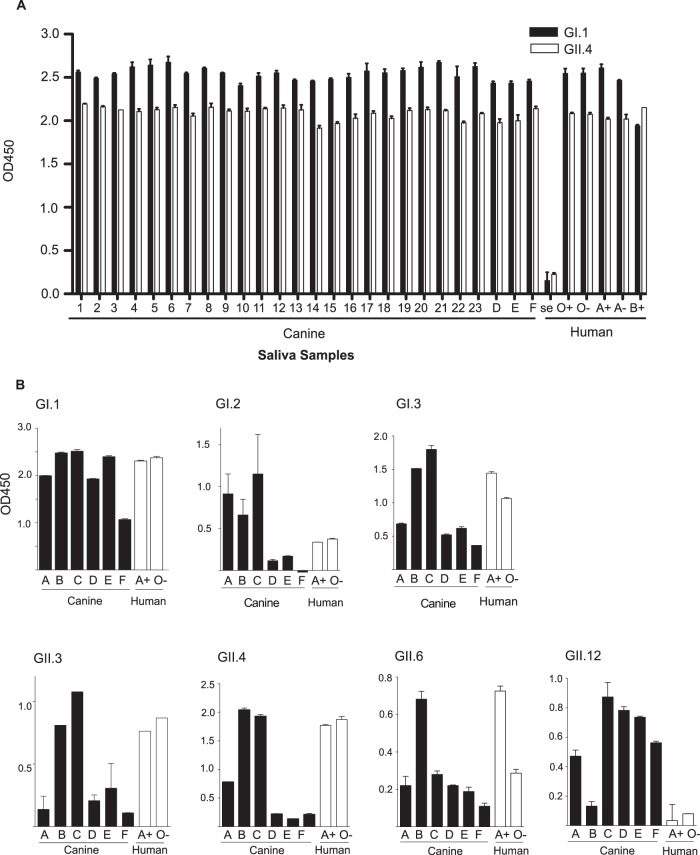
HuNoV binding to canine samples in ELISA-based assays. Saliva from 20 six dogs (A) and duodenal scrapings from six dogs (B) were analyzed to assess their ability to bind to HuNoV VLPs. GI.1 and GII.4 HuNoV VLPs were used to assess binding to both saliva and duodenal samples, and an additional five genotypes of HuNoV VLPs were used in the duodenal sample binding assays. Human saliva samples representing a range of HBGA phenotypes were used as positive and negative controls, i.e., secretor negative (se) or O/A/B antigen positive, with Lewis expression represented by +/−. All experiments were performed in duplicate, with error bars representing the standard error for each sample.

Saliva samples from 26 dogs (1 to 23 and D to F), and duodenal scrapings from 6 dogs (A to F) were analyzed in ELISA-based assays for their ability to bind to HuNoV VLPs (Fig. 2). All canine samples were phenotyped for HBGA expression in a previous study (23). It was therefore known that H antigen expression was present in every canine sample, and A antigen and Lewis antigen expression was polymorphic. Human saliva samples representing the major HBGA phenotypes present in humans were used as controls. These human samples included saliva from a nonsecretor individual (no HBGA expression) and saliva from humans expressing either A antigen, B antigen, or H antigen alone (O phenotype). Saliva samples with variation in Lewis antigen expression (+/−) were also included.

In the saliva binding assay (Fig. 2A), the nonsecretor human sample was unable to bind to HuNoV VLPs, as expected based on previous reports (18). In contrast, all canine saliva samples and all secretor human samples were able to bind to HuNoV GI.1 and GII.4 VLPs. There were comparable OD_450_ values for the canine and human saliva samples, indicative of similar levels of binding.

VLPs of seven different HuNoV genotypes were used to assess their ability to bind duodenal scrapings from six dogs (A to F) (Fig. 2B). Human saliva samples from an A antigen-positive, Lewis antigen-positive (A+) individual and an A antigen-negative, Lewis antigen-negative (O−) individual were used as positive controls; both samples were shown (Fig. 2A) to bind to GI.1 and GII.4 HuNoV VLPs. Figure 2B demonstrates that canine duodenal scrapings could bind to every HuNoV genotype tested. Individual variation between the samples was identified; for example, canine samples D, E, and F showed decreased binding to GI.2 and GII.4 HuNoV VLPs. Other dogs however, most notably dogs B and C, were able to bind to all HuNoV VLPs tested. This was not apparently related to HBGA phenotype; all dogs were H antigen positive, and dogs C and E were A antigen positive, whereas dogs A, B, D, and F were A antigen negative (as previously reported [23]). In addition, dogs were phenotyped for Lewis antigen, with dogs A and B being Lewis positive and the remainder Lewis negative (data not shown). Variation in OD_450_ scales between genotypes was arbitrary due to the primary antibody used; for detection of the GI VLPs, the primary antibody used had been raised against GI.1, whereas for the GII VLPs, the primary antibody was raised against GII.4.

### HuNoV VLPs bind to canine gastrointestinal tissue sections.

To determine whether HuNoV VLPs are able to bind to canine gastrointestinal tissues, fixed sections of duodenum from two dogs (B and C) were incubated with HuNoV VLPs for 1 h, and then immunohistochemistry (IHC) was used to detect binding of HuNoV VLPs to the tissue surface. As polymorphism for the A antigen is present in dogs (approximately 50% are A antigen positive [23]) and due to the known interaction between A antigen and HuNoV (32), HBGA phenotyping was also required. Confirmation of the presence or absence of H antigen and A antigen in the tissue sections used for the VLP binding was achieved by incubating the tissue sections with Ulex and anti-A antigen antibody, respectively, and IHC was performed. This demonstrated that dog C was A antigen positive and dog B was A antigen negative, hence enabling comparison of HuNoV VLP binding to canine samples representing the two major HBGA phenotypes. H antigen expression was not detectable in the A-positive dog, which is understood to be due to the ability of the A antigen to mask the H antigen, therefore preventing detection by Ulex lectin binding (33).

**FIG 3:**
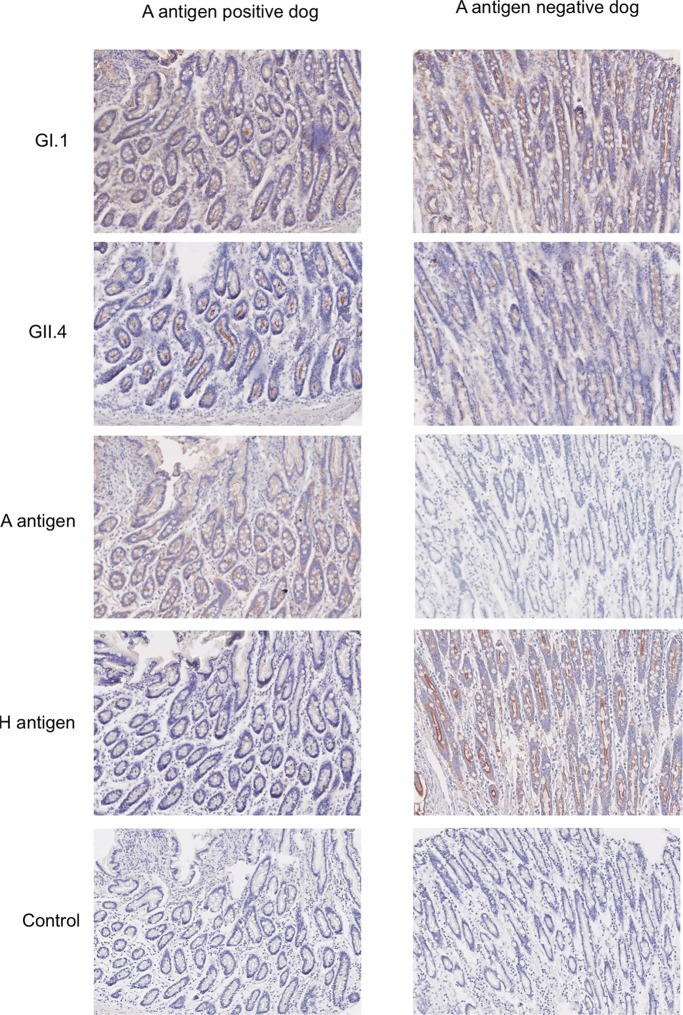
Binding of HuNoV VLPs to canine gastrointestinal tissue sections. HuNoV VLPs (GI.1 and GII.4) were incubated with tissue sections prior to staining for immunohistochemical analysis. A positive signal, either VLP binding or HBGA expression, is represented by red-brown staining. Two different canine phenotypes, i.e., a dog expressing A antigen (A positive), and a dog negative for A antigen expression, were compared, as presented previously (23).

Figure 3 demonstrates that GI.1 and GII.4 HuNoV VLPs can bind to both A antigen-positive and A antigen-negative dogs. In addition it was shown that HuNoV VLP binding has a pattern of expression similar to that of H and A antigen. Given the known interaction between HBGAs and HuNoVs, these similar binding patterns were expected (18).

### HuNoV RNA was not detected in canine stool samples.

A total of 248 canine stool samples were collected and analyzed for the presence of HuNoV RNA. Stool samples and clinical data were collected from 131 dogs admitted to veterinary clinics and a rescue kennel distributed across the United Kingdom between August 2012 and May 2014. The mean age of the dogs was 5.1 years (standard deviation, 4.3 years), with 56 different breeds represented. A total of 50.1% of these samples were from dogs with clinical signs of primary gastroenteritis. Control samples were collected from 117 healthy dogs (mean age, 5.6 years; standard deviation, 3.6 years) from boarding kennels or belonging to veterinary staff.

Nucleic acid extraction and qPCR were successfully performed on 248 stool samples as determined by constant threshold cycle (*C_T_*) values from the internal extraction control RNA. Samples were tested by qPCR for the presence of HuNoV and CNV. No samples were identified as being positive for any noroviruses, indicating that the overall prevalence of noroviruses in this population at the time of sample collection was <1.5% (Wilson binomial approximation; confidence interval, 95%). To confirm the efficacy of the screening method, samples were also tested for the presence of two additional canine viruses previously reported to be present in the United Kingdom, canine parvovirus (CPV) and canine enteric coronavirus (CECoV). Enteric viruses, either CPV or CECoV, were detected at high titer (>10^7^ copies/ml stool) in 17.9% (12/67) of dogs admitted with primary gastroenteritis. No viruses were detected at titers above the positive threshold of the assay in dogs without gastroenteritis.

### HuNoV-specific antibodies are present in dogs.

Seven genotypes of HuNoV VLPs were used in ELISAs to screen for anti-HuNoV antibodies in a total of 325 dogs. Serum samples were collected from two groups of dogs, i.e., 223 samples collected in 1999 to 2001 (cohort A) and 102 samples collected in 2012 to 2013 (cohort B). Three GI HuNoV VLPs (GI.1, GI.2, and GI.3) were pooled for preliminary assays, as were four GII VLPs (GII.3, GII.4, GII.6, and GII.12).

The primary anti-HuNoV antibody screen identified anti-HuNoV antibodies at detectable levels in sera from 43 dogs, 24 from cohort A (10.7%) and 19 from cohort B (18.6%) (Table 3). Of these 43 dogs, 32.5% were seropositive for both GI and GII HuNoV, whereas the remainder were seropositive for either GI or GII HuNoV. Seropositivity to CNV in the same canine serum samples has previously been reported (28), and these data have been added to Table 3 for comparison. The age of the dog at time of sampling was known for 93/102 dogs in cohort B. No relationship between seropositivity to HuNoV and age was identified (data not shown).

**TABLE 3:** Seroprevalence of canine and human noroviruses in two canine cohorts^*a*^

Yr of serum collection	No. of canine samples screened	No. (%) of sera:				
		HuNoV positive				CNV positive
		GI only	GII only	GI and GII	Total	
1999–2001	223	5 (2.2)	11 (4.9)	8 (3.6)	24 (10.7)	85 (38.1)
2012–2013	102	3 (2.9)	9 (8.8)	7 (6.9)	19 (18.6)	62 (60.8)
All	325	8 (2.5)	20 (6.2)	15 (4.6)	43 (13.2)	147 (45.2)

a Serum samples were screened in ELISAs against pooled VLPs. The HuNoV GI pool consisted of genotypes GI.1, GI.2, and GI.3. The HuNoV GII pool consisted of GII.3, GII.4, GII.6, and GII.12. The CNV pool consisted of strains 170, C33, and HK.

To estimate the magnitude of the canine anti-HuNoV antibody response, anti-HuNoV titers were determined for 21/23 samples seropositive to GI HuNoV and 33/35 samples seropositive to GII HuNoV. As presented in Table 4, the antibody titers to GI in the 21 dogs seropositive in the primary ELISA screen are relatively low, but the OD_450_ values obtained in the titer ELISA showed strong consistency in comparison with the original ELISA screen. For the majority of the anti-GII HuNoV-positive serum samples, a similarly low antibody titer (mode, 1:100) was determined, but in contrast to the case for GI, three samples (9% of GII-seropositive samples tested) had antibody titers of 1:800 or higher.

**TABLE 4:** Anti-HuNoV antibody titers in canine serum

Anti-HuNoV antibody titer	No. (%) of samples:	
	GI positive	GII positive
1:50	8 (38.1)	5 (15.2)
1:100	8 (38.1)	12 (36.4)
1:200	5 (23.8)	7 (21.2)
1:400	0	6 (18.2)
1:800	0	2 (6.1)
1:1,600	0	1 (3)

**FIG 4:**
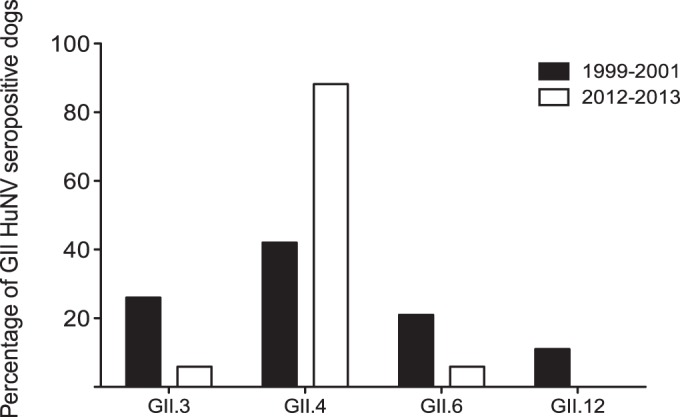
Genotype specificity of GII HuNoV-seropositive canine samples. Serum samples positive to pooled GII HuNoV were screened against GII.3, GII.4, GII.6, and GII.12 HuNoV VLPs individually. The genotype to which the highest OD_450_ reading was obtained was designated the primary genotype to which the antibody response was elicited. The proportions of GII HuNoV-positive samples from 1999 to 2001 (cohort A) and 2012 to 2013 (cohort B) reactive to each GII genotype tested were compared.

To extend the findings of the preliminary ELISAs, all canine serum samples positive for HuNoV were entered into a second round of ELISAs with individual genotypes of HuNoV. This was to investigate whether it was possible to identify the HuNoV genotype that may be eliciting the anti-HuNoV immune response. It is acknowledged that immunological cross-reactivity does exist between norovirus genotypes (34), and thus conclusive identification of the primary genotype inducing antibody production was not the aim of these experiments. However, the genotype to which the highest OD_450_ value was induced in ELISAs was tentatively suggested to be the major HuNoV genotype involved. For example, a serum sample for which the OD_450_ was highest against GII.4 HuNoV VLPs was designated GII.4 specific for the purposes of this study. Figure 4 presents the genotype distribution of HuNoV GII-positive samples, comparing cohort A (1999 to 2001) with cohort B (2012 to 2013). Our data showed that GII.4-specific antibodies were most common in both cohorts, although whereas 42.1% of samples showed the highest OD_450_ for GII.4 in cohort A, this figure increased to 87.5% in cohort B.

**FIG 5:**
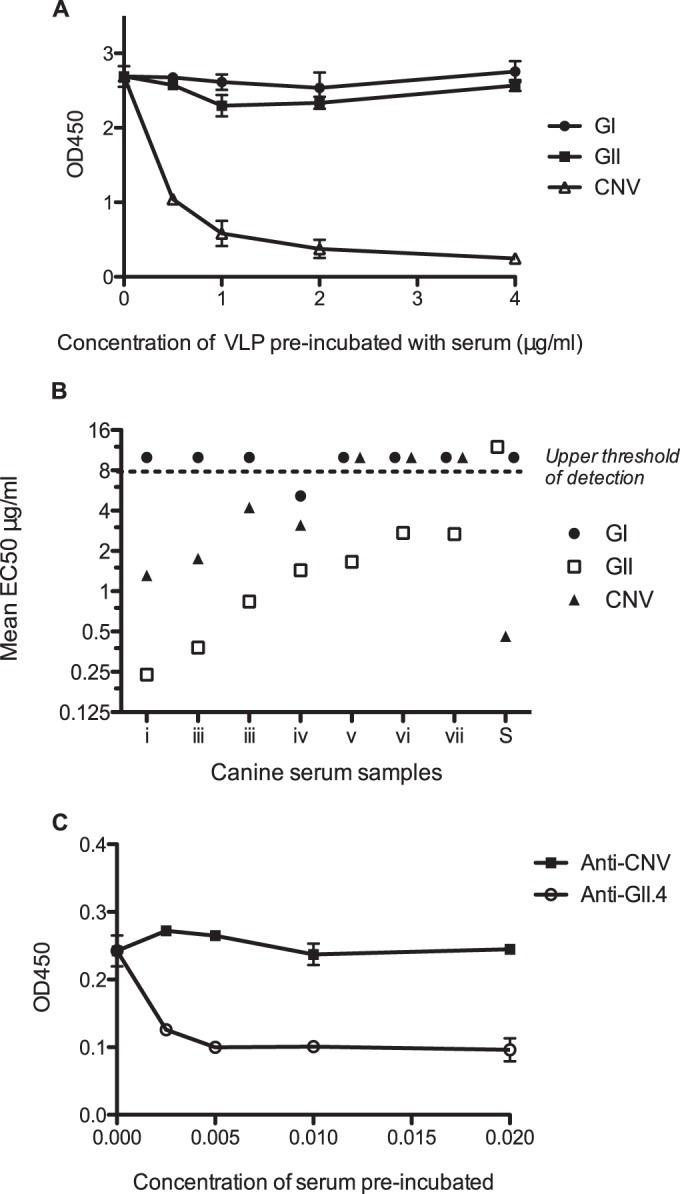
Evaluation of cross-reactivity between antibodies against human and canine noroviruses in canine sera. (A and B) VLP competition assays assessing the ability of canine sera to detect CNV (A) or GII.4 HuNoV (B) in the presence of alternative norovirus VLPs were conducted. (A) A representative CNV-positive canine serum was preincubated with serial dilutions of either pooled GI or GII HuNoV VLPs or pooled CNV VLPs, following the methodology previously presented (28). (B) Seven different GII.4 HuNoV-positive canine serum samples were preincubated with serial dilutions of either pooled GI or GII HuNoV VLPs or pooled CNV VLPs. The concentration of VLP required to block 50% binding (EC_50_) was calculated by fitting sigmoidal curves to the serial dilution data to allow comparison between serum samples. The dashed line represents the upper limit of detection. (C) An antibody competition assay was performed using antibodies specifically raised against CNV (rat) and GII.4 HuNoV (rabbit). Anti-CNV and anti-GII.4 antibodies were preincubated with GII.4 VLPs on a microtiter plate, and then after three plate washes, GII.4-positive canine serum was added and the OD_450_ of this interaction determined.

To confirm that the anti-HuNoV antibodies identified in dogs were not merely the result of cross-reactivity to canine-specific noroviruses, a series of blocking assays were performed (Fig. 5). As highlighted in Table 2, we have previously shown that seroprevalence to CNV was high in the same population of dogs analyzed in this study (28), so first it was necessary to establish that the CNV-specific antibodies were not cross-reactive with HuNoV. This was achieved by preincubating various concentrations of HuNoV and CNV VLPs with a representative anti-CNV antibody-positive canine serum (serum S) and then analyzing the ability of the serum to detect CNV VLPs (Fig. 5A). Preincubation with CNV VLPs was clearly able to block recognition of CNV VLPs by canine serum, whereas preincubation with GI or GII VLPs had no effect on CNV VLP recognition. This confirmed that the epitopes recognized by the anti-CNV antibodies were distinct from epitopes present on HuNoV VLPs.

Next, the specificity of the anti-GII antibodies identified in canine sera was examined using a similar VLP competition assay with GII VLPs applied to a microtiter plate instead of CNV VLPs (Fig. 5B). The concentration of HuNoV or CNV VLPs required to block 50% binding to GII VLPs was calculated by fitting a sigmoidal curve to the OD_450_ values for the serial dilution of VLPs. Seven different canine serum samples (i to vii) identified as being positive for anti-GII antibodies were analyzed, and serum S (negative for GII binding) was added as a negative control. For samples i to vii, the type of VLP inducing the lowest EC_50_ for blocking GII VLP recognition by canine serum was GII HuNoV VLPs. CNV VLPs did induce a decrease in GII recognition below the upper threshold of detection in 4/7 cases, but a greater concentration of CNV VLPs than of GII VLPs was required. This suggests that a degree of cross-reactivity between GII HuNoV and CNV is likely but that differentiation is possible.

The final blocking assay conducted to investigate the specificity of antibodies detected in canine serum used antibodies generated in animals immunized solely with either CNV or GII HuNoV VLPs (Fig. 5C). These animals, rat and rabbit, respectively, would not have been exposed to natural infection, and hence antibodies in their serum were deemed specific for their VLP immunogen. Anti-CNV or anti-GII HuNoV serum was serially diluted and incubated directly with GII VLPs applied to microtiter plates, and then after plate washing, GII-positive canine serum (serum vi) incubation followed. The results showed that rat CNV-specific antiserum was unable to block recognition of GII HuNoV by canine serum, whereas rabbit GII-specific antiserum induced blocking of GII VLP recognition by canine serum.

**FIG 6:**
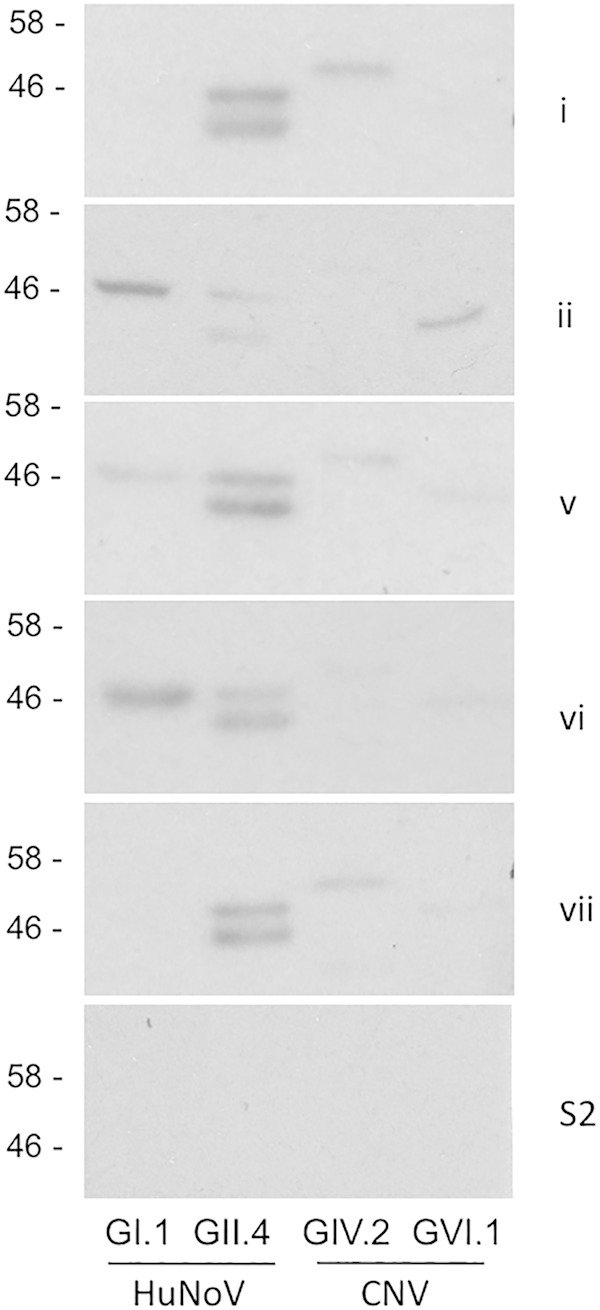
Western blotting of purified VLPs using seropositive serum. Norovirus VLPs from 4 genogroups, GI and GII (HuNoV) and GIV and GVI (CNV), were separated by SDS-PAGE. The polyacrylamide gel was then used for Western blotting with five different canine serum samples positive to GII.4 by ELISA and a single canine serum sample negative to all norovirus VLPs tested.

Western blotting was used as alternative method to demonstrate the presence of anti-HuNoV antibodies in canine sera. Five serum samples identified as being positive for anti-HuNoV antibodies by ELISA were selected for use in Western blots (Fig. 6). A single serum sample (sample S2) shown to be negative for both human and canine noroviruses was selected as a negative control. Western blotting confirmed that canine sera from five representative samples could detect GII.4 VLPs and that this expression was independent of recognition of genogroup IV or VI CNV.

## DISCUSSION

This study sought to investigate the likelihood that dogs can be infected with HuNoV, following initial reports that HuNoV can be detected in the stools of dogs (17). The results of our serological survey and VLP binding studies strongly suggest that dogs are susceptible to HuNoV. However, the frequency with which this occurs is deemed low based on the epidemiological results from this report. Furthermore, the clinical implications for both dogs and people in contact with dogs still remain to be confirmed.

In humans, it has been shown that HuNoVs bind to cell surface carbohydrates of the HBGA family prior to internalization. HBGAs are expressed on epithelial cells of many species, and we have recently confirmed that this includes dogs (23). This finding led us to hypothesize that HuNoV would be able to bind to the gastrointestinal tracts of dogs, and the ELISA and IHC data presented in this report were able to confirm this. This demonstrates that the initial step required for HuNoV entry into canine cells is present. However, it should be noted that rabbit hemorrhagic disease virus (RDHV), a related but distinct member of the Caliciviridae family, can bind to HBGAs (H type 2, A antigen and B antigen) (35), and yet there is no evidence RHDV can infect any species other than wild and domestic rabbits of the Oryctolagus cuniculus species. HBGA binding may be an initial step in calicivirus-host interaction, but a subsequent host-restrictive step(s) must be necessary for RHDV infection and potentially for HuNoV infection in dogs.

The viral RNA survey conducted as part of this project did not reveal any canine stool samples containing HuNoV RNA. This implies that the incidence of HuNoV shedding by this population of dogs is negligible, despite samples being collected from healthy dogs (117 animals), dogs with nongastroenteric disease (64 animals), and dogs with severe gastroenteritis requiring veterinary attention (67 animals). Inclusion of samples from the latter two groups was essential, as it has been suggested that HuNoV may be more likely to infect dogs with underlying disease or immunodeficiency (17), and as canine-specific noroviruses are associated with gastroenteritis in dogs (3, 36), it was hypothesized that HuNoV infection of dogs may cause signs of gastroenteric disease. Gastroenteritis is a common condition in dogs, with an owner questionnaire reporting diarrhea in 14.9% of dogs within the previous 2-week period (37) and 6% of canine veterinary consultations addressing gastroenteritis as a primary complaint (38). Of the 67 dogs with gastroenteritis in our survey, CPV (10 dogs) and CECoV (2 dogs) were detected in 17.9%. This proves that while viral gastroenteritis is relatively common in dogs, noroviruses are not a major cause of viral disease in the population of dogs sampled. The likelihood of HuNoV infection in a dog resulting in clinical signs of gastroenteritis is clearly much lower than that of CPV and CECoV infection, and as such, there is no immediate cause for concern by owners and veterinarians.

The absence of HuNoV-positive stool samples from dogs in this study is in contrast to the results of Summa et al. (17), who identified HuNoV RNA in 4/92 canine stool samples. However, their sampling strategy was significantly different from our approach; canine stool samples were collected only if the owner had shown symptoms of gastroenteritis within the past week, whereas stool samples in this study were collected with no reference to recent owner illness. HuNoV in humans is typically an acute infection, with peak viral shedding occurring 2 to 4 days after infection. By 3 weeks after infection, only 25% cases are still positive for viral RNA (39). In addition, although HuNoV is responsible for millions of infections worldwide each year, the virus is only identified in approximately 18% of human diarrheic samples submitted (40). Detection of HuNoV RNA in feces can be limited by factors such as low virus concentrations, improper storage of samples, inefficient viral RNA extraction, and the presence of fecal reverse transcriptase inhibitors (41). Overall, this indicates that positive identification of HuNoV shedding in dogs will be possible only within a very narrow time frame and that a proportion of cases will be false negatives. This suggests that in order to confirm that HuNoV can be shed in canine stools and to determine an accurate prevalence rate, a much larger sample size and/or a more focused sampling approach, e.g., collection of stool samples from owners with confirmed HuNoV infection, will be required.

Serological analysis of 325 canine serum samples in this study strongly suggests that dogs mount immune responses against HuNoV. We have demonstrated that almost 20% of dogs sampled in 2012 to 2013 had antibodies that could recognize HuNoV VLPs. This suggests that 1 in 5 dogs has been exposed to HuNoV in the United Kingdom. This proportion was lower than the proportion of dogs (43%) reported to be seropositive to HuNoV by a recent survey across Europe (16), which may be a reflection of population differences. An important conclusion from both studies is that the HuNoV seroprevalence rate identified in dogs is substantially lower than HuNoV seropositivity among human populations. In the United Kingdom nearly 100% of people are seropositive for GII.4 (42). This indicates that either dogs are exposed much more rarely to HuNoV or they are much less susceptible to infection than humans. Given that in one questionnaire-based study, 96% of dogs slept in their owners' houses and that when owners are at home almost 60% dogs were allowed anywhere in the house (43), it seems unlikely that dogs would not be exposed to HuNoV in a household with infected humans. Therefore, we propose that dogs are susceptible to HuNoV but at a much lower level than humans.

It could be argued that the anti-HuNoV antibodies identified in canine sera may have been generated in response to infection with related nonhuman noroviruses and are merely cross-reactive with HuNoV. For example, anti-CNV antibodies were detected in 45.2% of serum samples used in this study (28). To investigate this further, a series of blocking assays were performed using canine serum samples and serum samples from rats inoculated with CNV VLPs. These were able to show that the anti-GII HuNoV antibodies were specific for GII HuNoV VLPs and not three different strains of CNV (GIV and GVI). It is acknowledged that there are other nonhuman and noncanine noroviruses to which dogs may have been exposed, for example, swine, bovine, and feline noroviruses, cross-reactivity to which was not assessed. However, cross-reactivity between genogroups is known to be limited (34), and thus antibodies specific for feline noroviruses (GIV.2 genotype, the same as certain canine noroviruses) (44) or bovine noroviruses (GIII) are highly unlikely to cross-react with GII human noroviruses. Swine noroviruses, however, are classified into GII alongside human strains (45), and thus there is a greater risk of cross-reactivity. Nevertheless, due to United Kingdom farming practices, the frequency with which dogs in the study population would come into contact with pigs was deemed to be significantly lower than the frequency of contact with humans. In addition, although the feeding of raw pork to dogs does infrequently occur, animal noroviruses are extremely unlikely to be found in commercial pet food due to United Kingdom manufacturing processes and regulations (46).

The initial serosurvey demonstrated that dogs were more likely to be seropositive to GII HuNoV strains than to GI strains. This was in line with the findings from a recent European study (16). To explore this further, any HuNoV-positive samples were entered into a second round of ELISAs with VLPs from seven individual genotypes. This showed that the highest seroprevalence was to GII.4 strains. This is remarkable, as this is the most common genotype infecting humans worldwide. This also correlates with the report which identified HuNoV in the stools of four dogs (17). GII.4 HuNoV was detected by qPCR in the stools of 3 dogs and GII.12 in the stools of 1 dog.

Comparison of canine serum samples from two time periods (1999 to 2001 and 2012 to 2013) allowed analysis of the change in the prevalence of anti-HuNoV antibodies over time. Although the two study populations are not directly comparable (the earlier group was from a rehoming kennel and the later from a veterinary referral hospital) and the range of HuNoV strains studied was limited, it was shown that the proportion of dogs seropositive for HuNoV increased over this period. The prevalence of HuNoV in humans in United Kingdom has increased over a similar time period, from 6% in 1999 to approximately 16% in 2009 (47). It is possible to speculate that the rise in HuNoV seroprevalence in dogs from 1999 onwards is a reflection of the increased levels of infection in the human population.

Overall, this study supports the hypothesis that dogs can become infected with HuNoV. However, there are many questions still outstanding. First, it is unknown whether HuNoV infection has the potential to cause clinical disease in dogs. To answer this definitively, experimental studies will be required. Second, assuming that dogs can become infected with HuNoV, it is unknown whether dogs will shed virus in their stools in sufficient quantities to infect humans. It has been estimated that as few as 18 HuNoV particles may be sufficient to cause infection in humans (48), so it is likely that very low levels of shedding will be infectious. However, differences in the physiology of the canine and human gastrointestinal tracts (e.g., pH [49]) mean that it is possible that particle infectivity varies between the species. A third unanswered question is whether dogs play a significant role in the epidemiology of certain HuNoV outbreaks. The majority of HuNoV outbreaks do not occur in places where dogs are commonly found, e.g., outbreaks on cruise ships or in hospitals, but a role for dogs perpetuating outbreaks in communities cannot be ruled out. A final question is whether there is potential for dogs to be coinfected with CNV and HuNoV simultaneously. There is also concern than CNV may be zoonotic based on serological and receptor studies (23, 50); hence, CNV/HuNoV coinfections may also be possible in humans. If coinfections can occur, there would be a theoretical risk for recombination between virus strains, leading to generation of a novel norovirus. This may have altered virulence in canine and human hosts, and ongoing surveillance for such recombinants is deemed important.

In summary, whereas HuNoV infection of dogs has been shown to be theoretically possible, the risk of this causing significant clinical disease in dogs is believed to be very low. As for the potential for HuNoV infection being transmitted between dogs and their owners, this has yet to be established, though it is recommended that sensible hygiene precautions be taken around pets, especially when gastroenteritis in either humans or dogs is present in a household.

## Supplementary Material

Supplemental material

## References

[1] Tam C, Rodrigues L, Viviani L, Dodds J, Evans Hunter P, Gray J, Letley L, Rait G, Tompkins D, O'Brien S 2012 Longitudinal study of infectious intestinal disease in the UK (IID2 study): incidence in the community and presenting to general practice. Gut 61:69–77. doi:10.1136/gut.2011.238386. 21708822 PMC3230829

[2] Zheng D-P, Ando T, Fankhauser RL, Beard RS, Glass RI, Monroe SS 2006 Norovirus classification and proposed strain nomenclature. Virology 346:312–323. doi:10.1016/j.virol.2005.11.015. 16343580

[3] Mesquita JR, Barclay L, Nascimento MSJ, Vinjé J 2010 Novel norovirus in dogs with diarrhea. Emerg Infect Dis 16:980–982. doi:10.3201/eid1606.091861. 20507751 PMC3086253

[4] Tran TH, Trainor E, Nakagomi T, Cunliffe NA, Nakagomi O 2013 Molecular epidemiology of noroviruses associated with acute sporadic gastroenteritis in children: global distribution of genogroups, genotypes and GII.4 variants. J Clin Virol 56:185–193. doi:10.1016/j.jcv.2012.11.011. 23218993

[5] Glass R, Parashar U 2009 Norovirus gastroenteritis. N Engl J Med 361:1776–1785. doi:10.1056/NEJMra0804575. 19864676 PMC3880795

[6] Lopman BA, Adak GK, Reacher MH, Brown DWG 2003 Two epidemiologic patterns of norovirus outbreaks: surveillance in England and Wales, 1992-2000. Emerg Infect Dis 9:71–77. doi:10.3201/eid0901.020175. 12533284 PMC2873766

[7] Ahmed SF, Klena JD, Mostafa M, Dogantemur J, Middleton T, Hanson J, Sebeny PJ 2012 Viral gastroenteritis associated with genogroup II norovirus among U.S. military personnel in Turkey, 2009. PLoS One 7:e35791. doi:10.1371/journal.pone.0035791. 22606235 PMC3350499

[8] Mathijs E, Stals A, Baert L, Botteldoorn N, Denayer S, Mauroy A, Scipioni A, Daube G, Dierick K, Herman L, Van Coillie E, Uyttendaele M, Thiry E 2012 A review of known and hypothetical transmission routes for noroviruses. Food Environ Virol 4:131–152. doi:10.1007/s12560-012-9091-z. 23412887

[9] Scipioni A, Mauroy A, Vinjé J, Thiry E 2008 Animal noroviruses. Vet J 178:32–45. doi:10.1016/j.tvjl.2007.11.012. 18294883

[10] Mattison K, Shukla A, Cook A, Pollari F, Friendship R, Kelton D, Bidawid S, Farber JM 2007 Human noroviruses in swine and cattle. Emerg Infect Dis 13:1184–1188. doi:10.3201/eid1308.070005. 17953089 PMC2828081

[11] Chao D-Y, Wei J-Y, Chang W-F, Wang J, Wang L-C 2012 Detection of multiple genotypes of calicivirus infection in asymptomatic swine in Taiwan. Zoonoses Public Health 59:434–444. doi:10.1111/j.1863-2378.2012.01483.x. 22489630

[12] Farkas T, Nakajima S, Sugieda M, Deng X, Zhong W 2005 Seroprevalence of noroviruses in swine. J Clin Microbiol 43:657–661. doi:10.1128/JCM.43.2.657-661.2005. 15695660 PMC548037

[13] Cheetham S, Souza M, Meulia T, Grimes S, Han MG, Saif LJ 2006 Pathogenesis of a genogroup II human norovirus in gnotobiotic pigs. J Virol 80:10372–10381. doi:10.1128/JVI.00809-06. 17041218 PMC1641747

[14] Humphrey T, Cruickshank J, Cubitt W 1984 An outbreak of calicivirus associated gastroenteritis in an elderly persons home. A possible zoonosis? J Hyg (Lond) 92:293–299. 10.1017/s0022172400064822 PMC2129430 6094667

[15] Peasey AE, Ruiz-Palacios GM, Quigley M, Newsholme W, Martinez J, Rosales G, Jiang X, Blumenthal UJ 2004 Seroepidemiology and risk factors for sporadic norovirus/Mexico strain. J Infect Dis 189:2027–2036. doi:10.1086/386310. 15143470

[16] Mesquita JR, Delgado I, Costantini V, Heenemann K, Vahlenkamp TW, Vinjé J, Nascimento MSJ 2014 Seroprevalence of canine norovirus in 14 European countries. Clin Vaccine Immunol 21:898–900. doi:10.1128/CVI.00048-14. 24671552 PMC4054240

[17] Summa M, von Bonsdorff C.-H, Maunula L 2012 Pet dogs—a transmission route for human noroviruses? J Clin Virol 53:244–247. doi:10.1016/j.jcv.2011.12.014. 22244255

[18] Marionneau S, Ruvoën N, Le Moullac-Vaidye B, Clement M, Cailleau-Thomas A, Ruiz-Palacois G, Huang P, Jiang X, Le Pendu J 2002 Norwalk virus binds to histo-blood group antigens present on gastroduodenal epithelial cells of secretor individuals. Gastroenterology 122:1967–1977. doi:10.1053/gast.2002.33661. 12055602 PMC7172544

[19] Marionneau S, Cailleau-Thomas A, Rocher J, Le Moullac-Vaidye B, Ruvoën N, Clément M, Le Pendu J 2001 ABH and Lewis histo-blood group antigens, a model for the meaning of oligosaccharide diversity in the face of a changing world. Biochimie 83:565–573. doi:10.1016/S0300-9084(01)01321-9. 11522384

[20] Hutson AM, Airaud F, LePendu J, Estes MK, Atmar RL 2005 Norwalk virus infection associates with secretor status genotyped from sera. J Med Virol 77:116–120. doi:10.1002/jmv.20423. 16032732

[21] Lindesmith L, Moe C, Marionneau S, Ruvoen N, Jiang X, Lindblad L, Stewart P, LePendu J, Baric R 2003 Human susceptibility and resistance to Norwalk virus infection. Nat Med 9:548–553. doi:10.1038/nm860. 12692541

[22] Hutson AM, Atmar RL, Marcus DM, Estes MK 2003 Norwalk virus-like particle hemagglutination by binding to H histo-blood group antigens. J Virol 77:405–415. doi:10.1128/JVI.77.1.405-415.2003. 12477845 PMC140602

[23] Caddy S, Breiman A, le Pendu J, Goodfellow I 2014 Genogroup IV and VI canine noroviruses interact with histo-blood group antigens. J Virol 88:10377–10391. doi:10.1128/JVI.01008-14. 25008923 PMC4178834

[24] Murray JK, Browne WJ, Roberts MA, Whitmarsh A, Gruffydd-Jones TJ 2010 Number and ownership profiles of cats and dogs in the UK. Vet Rec 166:163–168. doi:10.1136/vr.b4712. 20139379

[25] Erles K, Toomey C, Brooks HW, Brownlie J 2003 Detection of a group 2 coronavirus in dogs with canine infectious respiratory disease. Virology 310:216–223. doi:10.1016/S0042-6822(03)00160-0. 12781709 PMC7126160

[26] Marionneau S, Airaud F, Bovin NV, Le Pendu J, Ruvoen-Clouet N 2005 Influence of the combined ABO, FUT2, and FUT3 polymorphism on susceptibility to Norwalk virus attachment. J Infect Dis 192:1071–1077. doi:10.1086/432546. 16107962

[27] Kageyama T, Kojima S, Shinohara M, Uchida K, Fukushi S, Hoshino FB, Takeda N, Katayama K 2003 Broadly reactive and highly sensitive assay for Norwalk-like viruses based on real-time quantitative reverse transcription-PCR. J Clin Microbiol 41:1548–1557. doi:10.1128/JCM.41.4.1548-1557.2003. 12682144 PMC153860

[28] Caddy S, Emmott E, El-Attar L, Mitchell J, de Rougemont A, Brownlie J, Goodfellow I 2013 Serological evidence for multiple strains of canine norovirus in the UK dog population. PLoS One 8:e81596. doi:10.1371/journal.pone.0081596. 24339947 PMC3855277

[29] De Rougemont A, Ruvoen-Clouet N, Simon B, Estienney M, Elie-Caille C, Aho S, Pothier P, Le Pendu J, Boireau W, Belliot G 2011 Qualitative and quantitative analysis of the binding of GII.4 norovirus variants onto human blood group antigens. J Virol 85:4057–4070. doi:10.1128/JVI.02077-10. 21345963 PMC3126233

[30] Belliot L, Noel JS, Li J, Seto Y, Humphrey CD, Ando T, Glass RI, Monroe SS 2001 Characterization of capsid genes, expressed in the baculovirus system, of three new genetically distinct strains of “Norwalk-like viruses.” J Clin Microbiol 39:4288–4295. 11724834 10.1128/JCM.39.12.4288-4295.2001 PMC88538

[31] Jiang X, Wang M, Graham DY, Estes MK 1992 Expression, self-assembly, and antigenicity of the Norwalk virus capsid protein. J Virol 66:6527–6532. 1328679 10.1128/jvi.66.11.6527-6532.1992 PMC240146

[32] Huang P, Farkas T, Marionneau S, Zhong W, Ruvoën-Clouet N, Morrow AL, Altaye M, Pickering LK, Newburg DS, LePendu J, Jiang X 2003 Noroviruses bind to human ABO, Lewis, and secretor histo-blood group antigens: identification of 4 distinct strain-specific patterns. J Infect Dis 188:19–31. doi:10.1086/375742. 12825167

[33] Nyström K, Le Gall-Reculé G, Grassi P, Abrantes J, Ruvoën-Clouet N, Le Moullac-Vaidye B, Lopes AM, Esteves PJ, Strive T, Marchandeau S, Dell A, Haslam SM, Le Pendu J 2011 Histo-blood group antigens act as attachment factors of rabbit hemorrhagic disease virus infection in a virus strain-dependent manner. PLoS Pathog 7:e1002188. doi:10.1371/journal.ppat.1002188. 21901093 PMC3161982

[34] Hansman GS, Natori K, Shirato-Horikoshi H, Ogawa S, Oka T, Katayama K, Tanaka T, Miyoshi T, Sakae K, Kobayashi S, Shinohara M, Uchida K, Sakurai N, Shinozaki K, Okada M, Seto Y, Kamata K, Nagata N, Tanaka K, Miyamura T, Takeda N 2006 Genetic and antigenic diversity among noroviruses. J Gen Virol 87:909–919. doi:10.1099/vir.0.81532-0. 16528040

[35] Ruvoën-Clouet N, Ganière JP, André-Fontaine G, Blanchard D, Le Pendu J 2000 Binding of rabbit hemorrhagic disease virus to antigens of the ABH histo-blood group family. J Virol 74:11950–11954. doi:10.1128/JVI.74.24.11950-11954.2000. 11090195 PMC112478

[36] Martella V, Lorusso E, Decaro N, Elia G, Radogna A, D'Abramo M, Desario C, Cavalli A, Corrente M, Camero M, Germinario CA, Bányai K, Di Martino B, Marsilio F, Carmichael LE, Buonavoglia C 2008 Detection and molecular characterization of a canine norovirus. Emerg Infect Dis 14:1306–1308. doi:10.3201/eid1408.080062. 18680664 PMC2600395

[37] Hubbard K, Skelly BJ, Mckelvie J, Wood JLN 2007 Risk of vomiting and diarrhoea in dogs. Vet Rec 161:755–757. doi:10.1136/vr.161.22.755. 18056013

[38] Jones PH, Dawson S, Gaskell RM, Coyne KP, Tierney A, Setzkorn C, Radford AD, Noble PJ 2014 Surveillance of diarrhoea in small animal practice through the Small Animal Veterinary Surveillance Network (SAVSNET). Vet J 201:412–418. doi:10.1016/j.tvjl.2014.05.044. 25011707

[39] Rockx B, De Wit M, Vennema H, Vinjé J, De Bruin E, Van Duynhoven Y, Koopmans M 2002 Natural history of human calicivirus infection: a prospective cohort study. Clin Infect Dis 35:246–253. doi:10.1086/341408. 12115089

[40] Ahmed SM, Hall AJ, Robinson AE, Verhoef L, Premkumar P, Parashar UD, Koopmans M, Lopman BA 2014 Global prevalence of norovirus in cases of gastroenteritis: a systematic review and meta-analysis. Lancet Infect Dis 14:725–730. doi:10.1016/S1473-3099(14)70767-4. 24981041 PMC8006533

[41] Patel MM, Widdowson M-A, Glass RI, Akazawa K, Vinjé J, Parashar UD 2008 Systematic literature review of role of noroviruses in sporadic gastroenteritis. Emerg Infect Dis 14:1224–1231. doi:10.3201/eid1408.071114. 18680645 PMC2600393

[42] Menon VK, George S, Aladin F, Nawaz S, Sarkar R, Lopman B, Gray JJ, Gomara MI, Kang G 2013 Comparison of age-stratified seroprevalence of antibodies against norovirus GII in India and the United Kingdom. PLoS One 8:e56239. doi:10.1371/journal.pone.0056239. 23437102 PMC3578856

[43] Westgarth C, Pinchbeck G, Bradshaw J, Dawson S, Gaskell R, Christley R 2008 Dog-human and dog-dog interactions of 260 dog-owning households in a community in Cheshire. Vet Rec 162:436–442. doi:10.1136/vr.162.14.436. 18390853

[44] Pinto P, Wang Q, Chen N, Dubovi EJ, Daniels JB, Millward LM, Buonavoglia C, Martella V, Saif LJ 2012 Discovery and genomic characterization of noroviruses from a gastroenteritis outbreak in domestic cats in the US. PLoS One 7:e32739. doi:10.1371/journal.pone.0032739. 22389721 PMC3289677

[45] Sugieda M, Nagaoka H, Kakishima Y, Ohshita T, Nakajima S 1998 Detection of Norwalk-like virus genes in the caecum contents of pigs. Arch Virol 143:1215–1221. doi:10.1007/s007050050369. 9687878

[46] Department for Environment, Food & Rural Affairs and Animal and Plant Health Agency. 2014 Using animal by-products to make pet food. DEFRA, APHA, London, United Kingdom.

[47] Tam CC, O'Brien SJ, Tompkins DS, Bolton FJ, Berry L, Dodds J, Choudhury D, Halstead F, Iturriza-Gómara M, Mather K, Rait G, Ridge A, Rodrigues LC, Wain J, Wood B, Gray JJ 2012 Changes in causes of acute gastroenteritis in the United Kingdom over 15 years: microbiologic findings from 2 prospective, population-based studies of infectious intestinal disease. Clin Infect Dis 54:1275–1286. doi:10.1093/cid/cis028. 22412058

[48] Teunis PFM, Moe CL, Liu P, Miller SE, Lindesmith L, Baric RS, Le Pendu J, Calderon RL 2008 Norwalk virus: how infectious is it? J Infect Dis 1476:1468–1476. doi:10.1002/jmv.21237. 18551613

[49] Lui CY, Amidon GL, Berardi RR, Fleisher D, Youngberg C, Dressman JB 1986 Comparison of gastrointestinal pH in dogs and humans: implications on the use of the beagle dog as a model for oral absorption in humans. J Pharm Sci 75:271–274. doi:10.1002/jps.2600750313. 3701609

[50] Mesquita JR, Costantini VP, Cannon JL, Lin S-C, Nascimento MS, Vinjé J 2013 Presence of antibodies against genogroup VI norovirus in humans. Virol J 10:176. doi:10.1186/1743-422X-10-176. 23735311 PMC3680240

[51] Scheltinga SA, Templeton KE, Beersma MFC, Claas ECJ 2005 Diagnosis of human metapneumovirus and rhinovirus in patients with respiratory tract infections by an internally controlled multiplex real-time RNA PCR. J Clin Virol 33:306–311. doi:10.1016/j.jcv.2004.08.021. 15994117 PMC7185544

