# Supplementary material for: Evidence for Human Norovirus Infection of Dogs in the United Kingdom
Source: J Clin Microbiol. 2015 May 14;53(6):1873–83. doi: 10.1128/JCM.02778-14 (PMC4432062; doi:10.1128/JCM.02778-14)
Supplement: Supplemental material [file supp_53_6_1873__index.html]

Supplemental material 

# Evidence for Human Norovirus Infection of Dogs in the United Kingdom

## Supplemental material

- Supplemental file 1 -

  Fig. S1 (Scatterplot of seropositivity to vesivirus 2117 and HuNoV)

  PDF, 126K
