# Supplementary material for: Evidence for Human Norovirus Infection of Dogs in the United Kingdom
Source: J Clin Microbiol. 2015 May 14;53(6):1873–83. doi: 10.1128/JCM.02778-14 (PMC4432062; doi:10.1128/JCM.02778-14)
Supplement: Supplemental material [file JCM.02778-14_zjm999094284so1.pdf]

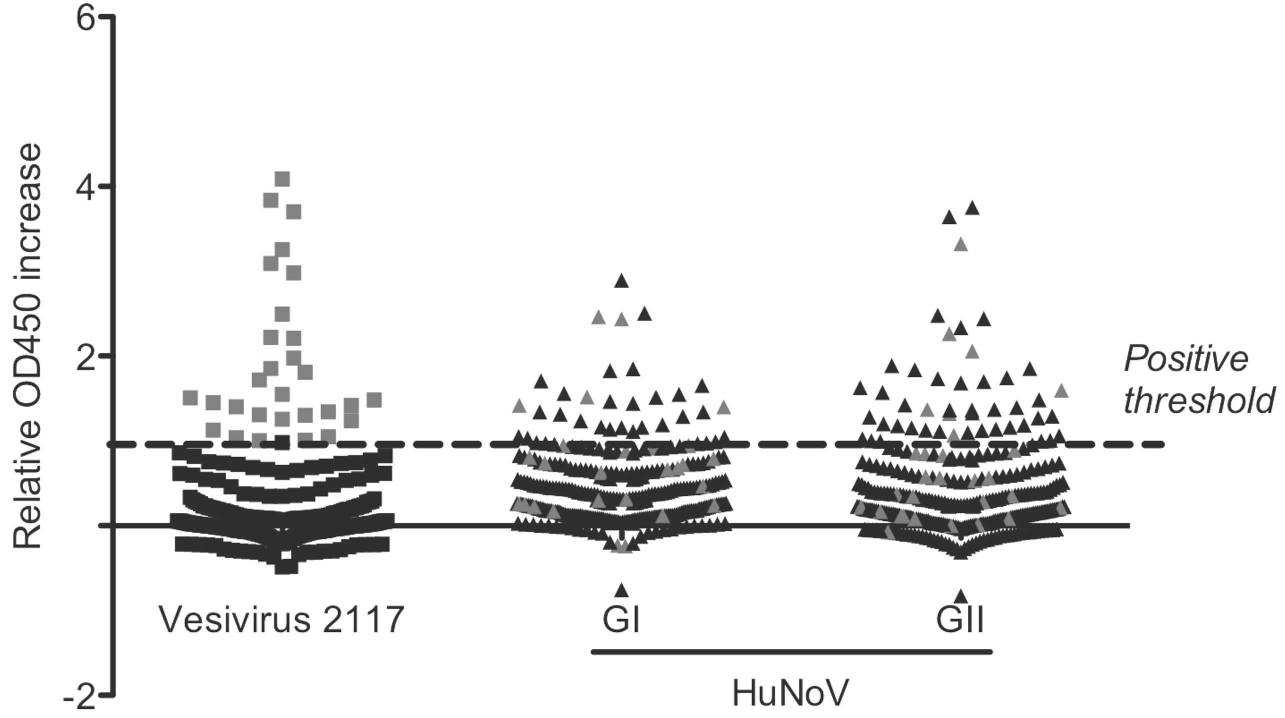

Figure S1. Scatterplot of seropositivity to vesivirus 2117 and HuNoV. Relative OD450 increase above the positive threshold for each plate was plotted to enable comparison between different ELISA plates. Dogs seropositive to vesivirus 2117 were identified if the relative increase in OD450 was greater than 1. The data points for these dogs are highlighted in grey. The relative OD450 increase to GI and GII HuNoV VLPs for every dog is plotted in the second and third scatter columns, with dogs seropositive to vesivirus 2117 again highlighted in grey.
